# The Impact of Digital Intervention Messages Targeting Users With High Blood Pressure Events: Retrospective Real-World Study

**DOI:** 10.2196/76275

**Published:** 2025-11-26

**Authors:** Yifat Fundoiano-Hershcovitz, Inbar Breuer Asher, Marilyn D Ritholz, David L Horwitz, Omar Manejwala, Claudia Levi, Pavel Goldstein

**Affiliations:** 1 DarioHealth, Hatarshish 5, North Industrial Park, Caesarea, Israel, 972-47704044; 2 Joslin Diabetes Center, Harvard Medical School, Boston, MA, United States; 3 DLH Biomedical Consulting, Las Vegas, NV, United States; 4 School of Public Health, University of Haifa, Haifa, Israel

**Keywords:** digital health intervention, blood pressure, hypertension, retrospective study, lifestyle behaviors

## Abstract

**Background:**

Effective hypertension management, particularly through self-care strategies, remains a significant public health challenge. Despite widespread awareness, only approximately 1 in 5 adults achieves adequate blood pressure (BP) control. There is a growing need for scalable digital health interventions that enhance awareness, support behavioral change, and improve clinical outcomes. However, real-world evidence evaluating the impact of such interventions on BP levels and their underlying mechanisms is limited.

**Objective:**

This study aimed to evaluate the effectiveness of a digital intervention using data-driven nudges on monthly average BP levels. Specifically, we assessed changes in BP before and after the intervention and examined whether these changes differed compared to a control group in a high BP cohort and a normal BP cohort.

**Methods:**

In this retrospective, real-world cohort study, we analyzed two user cohorts from a digital health platform: (1) individuals with high BP readings and (2) individuals with normal BP readings. Participants who received a digital intervention were propensity score–matched to users who did not receive the intervention, based on demographic and clinical variables. Monthly average BP and the proportion of high readings were assessed 3 months before and after the intervention. A piecewise mixed-effects model was used to evaluate BP trajectories, and simple slope analysis assessed the interaction between the outcomes and the groups, as well as the moderating effect of lifestyle activities on systolic blood pressure (SBP).

**Results:**

In total, 408 users were included in the study. In the high BP cohort (n=296), the intervention group showed a significant decrease in the monthly average SBP after the intervention (B=–2.09; *P*<.001), while the control group showed a smaller reduction (B=–1.06; *P*=.007). Additionally, users reporting higher lifestyle activity levels experienced a greater reduction in SBP (B=–5.27; *P*<.001). In the normal BP cohort (n=112), the intervention group maintained stable BP levels after the intervention (B=–0.39; *P*=.27), while the control group exhibited a significant increase in BP levels (B=0.69; *P*=.03).

**Conclusions:**

Data-driven nudges delivered via a digital health platform were associated with improved BP outcomes among individuals with high BP levels and helped maintain BP stability among those with normal BP levels. These findings reinforce the integration of personalized digital interventions into hypertension management and highlight the potential role of positive messaging, behavioral engagement, and user empowerment in improving long-term outcomes.

## Introduction

High blood pressure (BP) is a major public health challenge [1], but it has also been identified as the leading preventable risk factor for premature death [2]. Hypertension has been diagnosed in approximately 1.4 billion (31%) adults aged 30 to 79 years worldwide [3] and 108 million adults in the United States [4]. The prevalence of hypertension is rising globally owing to the aging of the population and an increase in the exposure to lifestyle risk factors [3] such as an unhealthy diet and sedentary lifestyle [5]. Additionally, the implementation of new guidelines in 2017, which lowered the diagnostic threshold for hypertension to systolic blood pressure (SBP) ≥130 mm Hg and diastolic blood pressure (DBP) ≥90 mm Hg [6], has led to more people being classified as hypertensive.

In addition, high BP is controlled in only about 1 in 5 adults (21%) [7,8], defined as maintaining SBP below 140 mm Hg and DBP below 90 mm Hg, according to the Joint National Committee (JNC7) guidelines. Furthermore, there are substantial disparities in disease awareness, treatment, and control across different racial and ethnic groups in the United States [9]. Many factors contribute to these inequalities, such as health literacy, socioeconomic status, reduced access to healthy foods, and health literacy [9]. These disparities impact the overall burden of hypertension in the United States and highlight the need for health care strategies to address hypertension across all populations.

Hypertension is a chronic condition that fluctuates over time, with periods of stability interrupted by episodes of elevated pressure. This variability is one of the reasons why hypertension is so challenging to manage. Users may experience fluctuations that complicate diagnosis and long-term management [10]. Monitoring BP regularly is key to minimizing these ups and downs and helping prevent the complications associated with chronic hypertension [11,12].

Treatment and management of hypertension are critically important for the reduction of cardiovascular complications and for the prevention of consequent diseases [13,14]. Despite the proven efficacy of pharmacological treatments [15] and the effectiveness of the variety of nonpharmacological interventions in lowering BP [16], poor BP control remains a pervasive problem. Suboptimal adherence, characterized by the failure to initiate treatment and to persist in therapy in the long term, is a well-recognized factor contributing to the inadequate control of BP in hypertension [17]. Studies have shown that low adherence is associated with reduced therapeutic success, reduced quality of life, and higher treatment costs [18].

Numerous factors can influence adherence in users with hypertension, including older age, lower education levels, potential medication side effects, and insufficient guidance from health care professionals [19]. Additionally, low adherence may stem from the common misconception among users that medication is unnecessary, as hypertension often presents without symptoms [19]. This highlights the importance of user education in managing hypertension, as it empowers them to make informed choices and effectively control risk factors, which can ultimately improve long-term health outcomes.

Health technology has created new opportunities to improve the management and treatment of chronic conditions like hypertension [20], particularly after the COVID-19 pandemic. Self-management education and support have been widely used as strategies aiming to provide users with the appropriate health literacy and skills for the effective, long-term control of hypertension [21]. In recent years, due to the fact that 86% of the global population has access to a smartphone, digital interventions allow a more convenient and accessible form of health care delivery, resulting in effective hypertension self-management [21].

Several studies have assessed the benefits of mobile health (mHealth) in promoting BP self-management and its effectiveness in managing other cardiometabolic conditions [17,22,23]. Digital tools provide a promising, cost-effective, and scalable solution to improve and sustain hypertension outcomes on a large scale. Meta-analyses have demonstrated that mHealth interventions not only reduce BP but also increase the reach, uptake, and feasibility of hypertension management [21]. Digital solutions for hypertension must be evidence-based and effective to reduce its impact on global noncommunicable diseases [24].

Progress in mHealth technology has enabled the design of just-in-time adaptive interventions (JITAIs) [25]. JITAIs have emerged as impactful approaches, providing support for behavior change and hypertension monitoring [26]. This type of intervention enables the delivery of real-time support and provides personalized contextual feedback. Despite the significant advancements in technology and the appeal of JITAIs, many programs have been developed with little empirical evidence, and research has been limited by a lack of evidence regarding effectiveness and sustained engagement [25,26].

Our study performed a retrospective analysis of a digital health platform for hypertension management by integrating a home-use BP-monitoring system with comprehensive data captured through a supportive mobile app for individuals with normal and poorly controlled BP levels. The study aimed to assess how a BP digital intervention with data-driven nudges would influence monthly average BP levels, with measurements of BP 3 months before and after the nudges. We hypothesized that prior to the delivery of the nudges, the 2 groups would exhibit similar BP levels. However, following the delivery of the nudges, the groups would show distinct trajectories in their BP levels. Specifically, we expected that the group receiving the nudges would demonstrate a greater reduction in their BP compared to the group not receiving the nudges. By analyzing the 2-stage period, we aimed to gain a comprehensive understanding of the impact of educative messages and positive feedback, assessing whether changes occurred between the pre- and postintervention phases and whether these changes differed between the 2 groups.

## Methods

### Platform

This study used the Dario Health digital health platform to support the self-management of hypertension within the context of chronic cardiometabolic conditions. The Dario BP monitoring system combines a connected BP monitor with a mobile app (compatible with both Android and iOS devices). The BP-monitoring system measures the SBP, DBP, and pulse rate by using a noninvasive technique in which an inflatable cuff is wrapped around the upper arm. Viewing both pulse rate and BP provides a more comprehensive picture of cardiovascular health. These 2 metrics can impact each other in different ways and offer valuable insights into a user’s overall cardiovascular health. For example, an unexpected combination of high or low readings can be a key indicator of underlying health issues. Similarly, a normal BP paired with a low resting pulse rate often indicates strong cardiovascular fitness [27,28]. The BP-monitoring system uses Bluetooth. The BP cuff is paired with the mobile app, and the data are transmitted to the smart mobile device via Bluetooth, ensuring real-time feedback and 100% data capture. The BP reading is displayed on the mobile app screen. The immediate display of measurements on the smartphone interface supports timely decision-making and enhances engagement.

Users can input additional data at the time of measurement, such as the arm used, recent activities (eg, smoking, caffeine intake, and physical activity), and symptoms (eg, stress, dizziness, and headache). This information is securely stored in a digital logbook, with automatic cloud backup enabling further analysis and clinical interventions tailored to individual needs.

The platform’s data-driven approach uses real-time nudges designed to improve health outcomes. These messages provide personalized feedback and educational inputs based on BP thresholds aligned with American Heart Association (AHA) guidelines, which provide standardized targets for BP management across all nonpregnant adults, regardless of age or gender [29]. Personalization is achieved through dynamic tailoring of messages based on each user’s clinical profile and real-time data trends. The system analyzes users’ longitudinal BP patterns and identifies trends, delivering in-app notifications, push messages, emails, and SMS text message alerts with actionable insights. When BP readings indicate specific trends, users receive targeted educational content, motivational messages, and behavioral prompts to encourage adherence to health goals and increase platform engagement. Specifically, the system analyzes users’ BP patterns to identify meaningful changes or persistently elevated readings. Messages are then customized according to these trends, offering feedback relevant to the user’s current status, such as positive reinforcement for sustained control or motivational nudges when elevated values are detected. For example, a user with persistently high SBP readings may receive targeted lifestyle suggestions (eg, sodium reduction and physical activity prompts), while a user demonstrating improvement may receive encouraging messages reinforcing adherence.

Three independent readings over 3 individual days (nonconsecutive) within a 7-day period will trigger a digital intervention, except for a hypertensive crisis, which causes a trigger for each event. The BP levels embedded in the system are similar to those defined by the AHA [29]: normal BP, SBP <120 mm Hg and DBP <80 mm Hg; elevated BP, SBP 120‐129 mm Hg and DBP <80 mm Hg; hypertension stage 1, SBP 130‐139 mm Hg or DBP 80‐89 mm Hg; hypertension stage 2: SBP ≥140 mm Hg or DBP ≥90 mm Hg; and hypertensive crisis, SBP >180 mm Hg and/or DBP >120 mm Hg.

**Figure 1.**
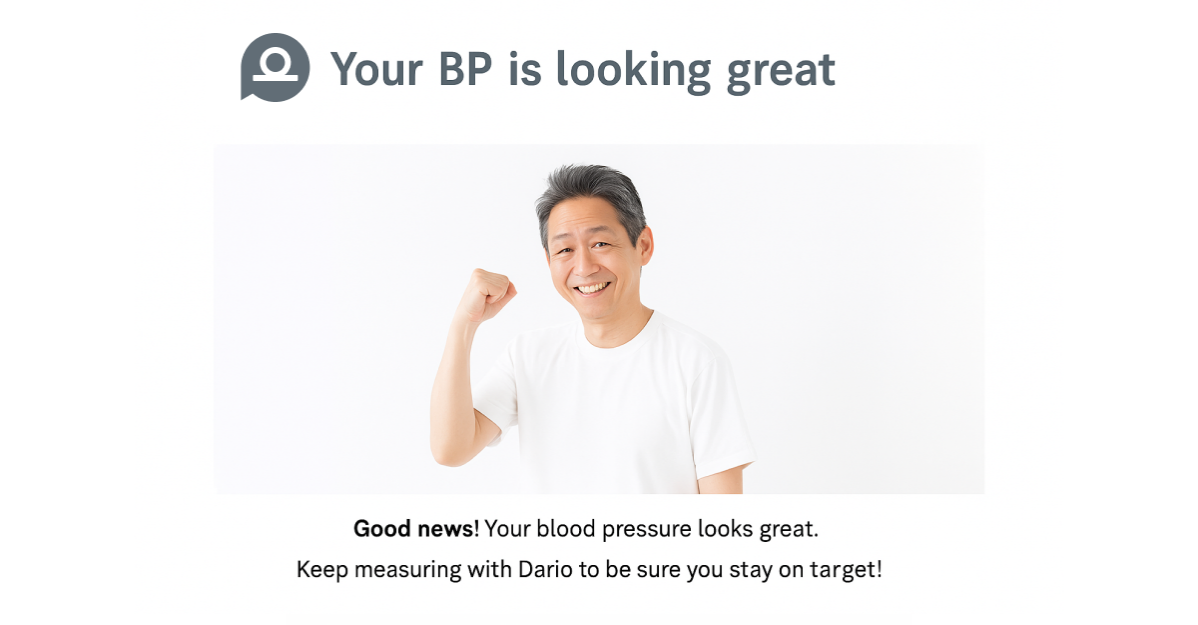
Representative intervention message delivered via the Dario app following normal blood pressure readings.

For normal BP clusters, motivational feedback was delivered via SMS text messages and in-app messages encouraging the user to keep measuring their BP to stay on target. The nudges triggered by normal BP levels included the following: “Well done! Your BP is looking good,” “Good news. Your blood pressure looks good,” “Keep measuring with Dario to be sure you stay on target!,” and “Your BP is looking great.” A representative message is presented in Figure 1.

For stage 1 hypertension, users received informational push notifications, SMS text messages, and educational articles through cluster events. Messaging was nonjudgmental in tone and included the following: “Your blood pressure readings are higher than normal, according to your numbers in the Dario App. Are you measuring correctly? Review the steps for measuring at home,” “Have you heard about the DASH diet? Link to DASH ref Learn more about the diet plan that has been found to help lower blood pressure,” and “Recent measurements show that your blood pressure is still higher than normal. If you’re taking your blood pressure medication as directed, it’s time to schedule a medication review. Want to make sure you are taking your medications correctly? Review a list of things you need to know.”

Educational topics were based on current hypertension management guidelines that recommend, as an integral part of ongoing treatment, the adoption of lifestyle modifications, including a healthy diet, independently of the underlying antihypertensive drug treatment [29]. Research has provided lifestyle recommendations, including a low-sodium, increased-potassium, low-fat diet; maintenance of appropriate body weight; alcohol use reduction; smoking cessation; increased physical activity; and stress reduction [30]. The average impact of each lifestyle change is a decrease of 4‐5 mm Hg in SBP and a decrease of 2‐4 mm Hg in DBP; however, a diet low in sodium, saturated fat, and total fat and an increase in the consumption of fruits, vegetables, and grains may decrease SBP by approximately 11 mm Hg [29]. Educational content includes general information on the meaning of BP values, the adoption of a healthy lifestyle or diet, and adherence to medication. Lifestyle changes include moving toward a healthy body weight, performing physical activity of 30 minutes a day, eating a heart-healthy diet, reducing alcohol consumption, quitting smoking, managing stress, and regularly monitoring BP [31].

DASH (Dietary Approaches to Stop Hypertension) studies have shown that diets rich in fruits and vegetables and low in saturated and total fats can both lower the risk of high BP and assist with BP control in people with hypertension. Vegetables and fruits account for approximately half of the BP-lowering effect of the diet. Foods in the DASH diet are rich in minerals such as potassium, calcium, and magnesium. The diet limits foods that are high in sodium. It also limits added sugar and saturated fat, such as that in fatty meats and full-fat dairy products. The standard DASH diet limits salt intake to 2300 mg a day. This amount agrees with the Dietary Guidelines for Americans [32]. A lower-sodium version of the DASH diet restricts sodium to 1500 mg a day. Restricting sodium intake can enhance the BP-lowering effect. While the DASH diet can reduce SBP by 5‐6 mm Hg, individuals eating the DASH diet in combination with the lowest sodium intake have been reported to achieve a further BP decrease of 7.1 mm Hg [33]. Compliance with prescribed therapies is a pivotal factor in treatment success. According to the World Health Organization, medication adherence can have a more direct impact on clinical outcomes than the specific treatment itself. Multiple factors contribute to adherence levels, stemming from individual, provider, and health care system elements, which often interact with each other. In the United States, only 51% of users adhere to their medication regimen for high BP [34,35]. Through the messages of the intervention, users also receive guidance on proper medication use to enhance adherence.

**Figure 2.**
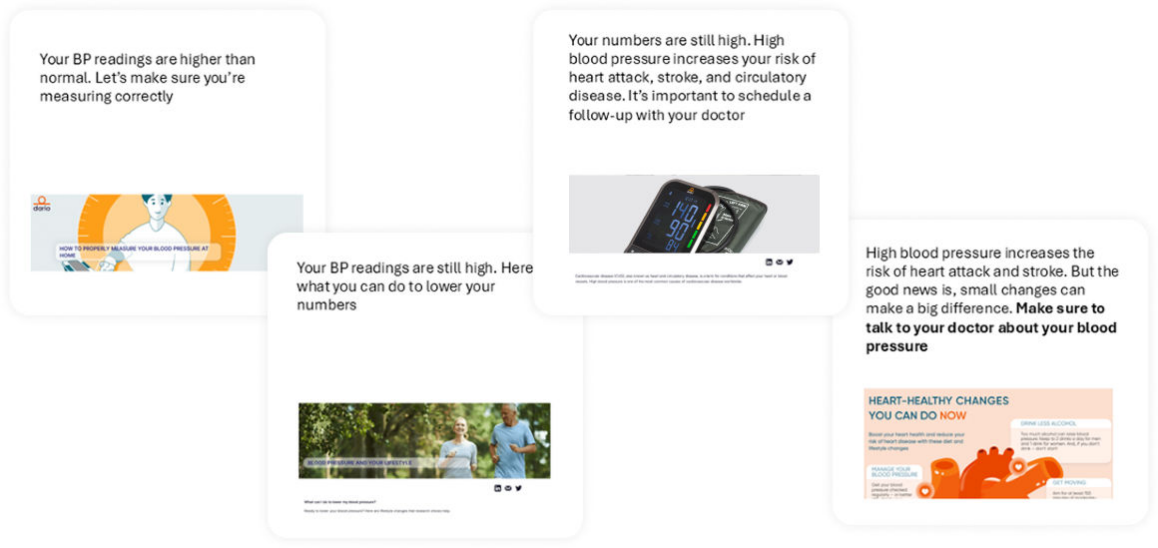
Representative intervention messages delivered via the Dario app following high blood pressure readings.

Representative messages for high reading clusters are presented in Figure 2.

### Measures

The monthly average BP level (SBP and DBP), which was defined as the mean of a user’s BP measurements taken over a 30-day period from the first nudge message, was used as a core outcome metric. Monthly aggregation was specifically chosen based on several evidence-based considerations: (1) it aligns with clinical practice guidelines that recommend assessing BP control trends over weeks to months rather than days [29]; (2) it provides sufficient statistical power to detect clinically meaningful changes while reducing noise from day-to-day physiological variability, which can be as high as 10‐15 mm Hg due to factors unrelated to intervention effects [12]; (3) it captures the sustained behavioral change trajectory that digital interventions aim to achieve, as behavior modification typically requires 3‐4 weeks to establish new habits [36]; and (4) it corresponds to the temporal scale at which medication adjustments and lifestyle interventions are evaluated in clinical trials, facilitating comparison with existing literature.

The monthly high-reading percentage, which was defined as the monthly number of high readings (SBP ≥140 mm Hg and DBP ≥90 mm Hg) divided by the monthly number of all BP measurements taken over a 30-day period from the first nudge message, was used as another core outcome metric. The mobile platform collected the following medical and sociodemographic information (by self-report) for each user: gender, age, weight, BMI, physical activity level, stress level (0 [no stress] to 10 [very stressed]), alcohol consumption (number of drinks per week), smoking (0 [never] to 3 [yes]), and comorbidities (eg, baseline hypertension [based on the first 30-day measurements on the platform], diabetes, high lipid levels, chronic kidney disease, cardiovascular disease, and cancer). Independent variables included digital engagement, such as the number of monthly BP measurements, and lifestyle activities (operationalized as the sum of meal logs, carbohydrate intake, calories burned, and recipe finder activities in each month).

### Study Population

A retrospective data study was performed on the Dario database. Individuals who used the Dario platform between 2019 and 2024 were considered. The users purchased the device via a direct-to-consumer channel. The study analyzed 2 cohorts of users who received a digital intervention (intervention group): one cohort of users who had high BP readings and another cohort of users who had normal BP readings [29]. The inclusion criterion for the intervention group (both cohorts) was measurement of BP using the Dario Health platform during the years 2021‐2024, for a minimum of 2 months (1 month prior to the first nudge message and 1 month after). To establish a control group, we selected users from the existing Dario database who never received a nudge between August 2019 and May 2020, and who experienced the same BP events. We applied the propensity score matching procedure to ensure comparability. The events that captured and triggered a digital intervention included high BP events (defined as SBP ≥130 mm Hg or DBP ≥80 mm Hg) occurring 4 times on different days within a 7-day period in the high BP cohort, and normal BP events (defined as SBP <120 mm Hg or DBP <80 mm Hg) occurring 4 times on different days within a 7-day period in the normal BP cohort.

### Ethical Considerations

All data used for the analysis were anonymized before extraction for this study. The study received an exemption from Ethical and Independent Review Services (a professional review board), which issued the institutional review board exemption (number: 18,032‐07#) [37]. The users who participated in the study were provided with a Terms of Use document mentioning the legally valid consent of the end user for the company to collect and access their information. The use of the app, site, or services is deemed to constitute user consent that is legally bound by the Terms of Use and the Privacy Policy. The Terms of Use do not specify an option for users to opt out of the use of their deidentified data for research purposes while continuing to use the service. The current Terms of Use can be accessed on DarioHealth [38]. No compensation was provided to users.

### Study Design

The aim of our study was to evaluate the impact of digital interventions on BP levels and assess their relative contribution to BP levels. We conducted 2 separate parallel analyses: one for the high BP cohort and another for the normal BP cohort. For the digital intervention group, it was crucial to establish a clear starting point for the intervention to assess its effects accurately.

All users in the intervention and control groups had access to the same Dario platform features, including BP monitoring, educational materials, and lifestyle tracking tools. The sole difference was that the intervention group received automated, personalized nudge messages triggered by their BP patterns, while the control group did not receive these triggered messages despite experiencing the same BP events.

This approach ensured that any observed differences in outcomes would be attributable to the digital intervention itself, rather than temporal factors or external influences. Using this approach, we enhanced the internal validity of the study. This allowed us to isolate the effect of the digital intervention from that of other variables and assess the impact on BP levels more accurately.

### Propensity Scores: Causal Inference

Propensity score matching was used in this study to address potential confounding factors and enhance the comparability of the intervention and control groups. The rationale behind using propensity score matching lies in its ability to reduce bias and mimic the randomization process, thereby facilitating causal inference in observational studies [39].

### Missing Data Assessment and Handling

Prior to the analysis, we conducted a comprehensive assessment of missing data patterns and mechanisms. The Little missing completely at random test [40] was performed, and there was no evidence against the missing completely at random assumption (*χ*^2^_11_=16.438; *P*=.13). Missing data were present only in baseline covariates used for propensity score matching, while outcome variables (BP measurements) had almost complete data at the month-aggregated level.

The missing data in both datasets were imputed using the Multivariate Imputation by Chained Equations (MICE) algorithm with the “mice” package in R (R Project for Statistical Computing). Specifically, the method used for imputation was predictive mean matching, with 5 imputed datasets generated (m=5) and a maximum of 50 iterations (maxit=50) for convergence. This approach allows for the generation of plausible values for missing data based on observed relationships in the dataset, ensuring that the imputed data preserve the underlying statistical structure.

Propensity score matching was used to estimate intervention effects while removing the bias created by the intervention covariates [41] and forming matched sets of treated and untreated individuals who share a similar value of the propensity score. The goal of propensity score matching is to simulate the conditions of a randomized controlled trial, creating a balance between groups in the distributions of covariates.

The matching [41-44] for the high BP cohort was based on the following sociodemographic and clinical parameters: age, gender, weight, baseline hypertension as a binary variable (based on the first 30-day measurements on the platform), number of comorbidities, diabetes type (type 2, prediabetes, or others), mean number of BP measurements, average SBP and DBP in the months before the messages were sent, and average SBP and DBP as well as mean high-reading percentage in the month the messages were sent. The matching for the normal BP cohort was based on the following sociodemographic and clinical parameters: age, gender, weight, baseline hypertension as a binary variable (based on the first 30-day measurements on the platform), number of comorbidities, diabetes type (type 2, prediabetes, or others), number of BP measurements, average SBP and DBP in the months before the messages were sent, and the first SBP and DBP measurements in each individual.

**Figure 3.**
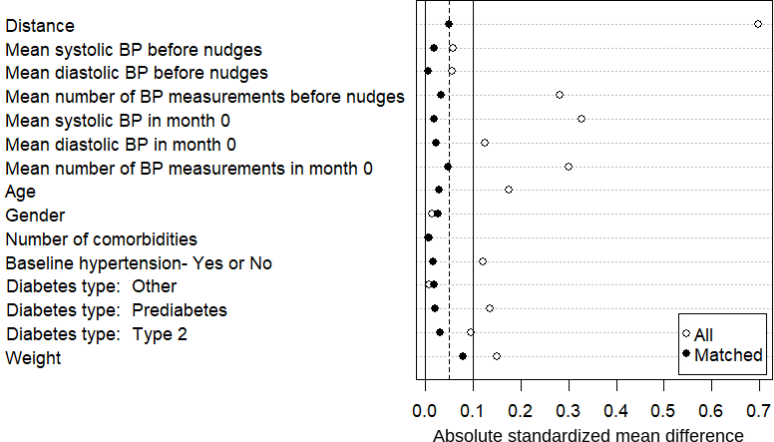
Plot presenting the efficacy of the matching procedure for balancing the high blood pressure (BP) cohort. Distance refers to the standardized difference in the propensity scores between the treatment and the control after matching. A caliper of 0.1 SD is reached for all parameters.

We used nearest-neighbor matching without replacement to ensure optimal balance between groups while maintaining interpretability. This approach was chosen over other methods (eg, full matching and weighting) for the following reasons: (1) it provides the most intuitive comparison between matched pairs, (2) it performs well when the overlap in propensity scores is substantial, and (3) it allows for straightforward assessment of covariate balance. A caliper width of 0.1 SDs of the logit of the propensity score was selected based on empirical evidence from Austin [43], who demonstrated that calipers of 0.2 SD eliminate approximately 99% of the bias due to measured confounders. We chose a more stringent caliper of 0.1 SD to further minimize potential bias while monitoring the trade-off with sample size reduction. Postmatching balance was assessed using standardized mean difference (SMD) for all covariates, with SMD <0.1 considered indicative of good balance [42]. As shown in Figures3  4, all covariates achieved SMD <0.1 after matching, confirming the effectiveness of our matching procedure. Additionally, we examined the distribution of propensity scores before and after matching to ensure adequate overlap.

**Figure 4.**
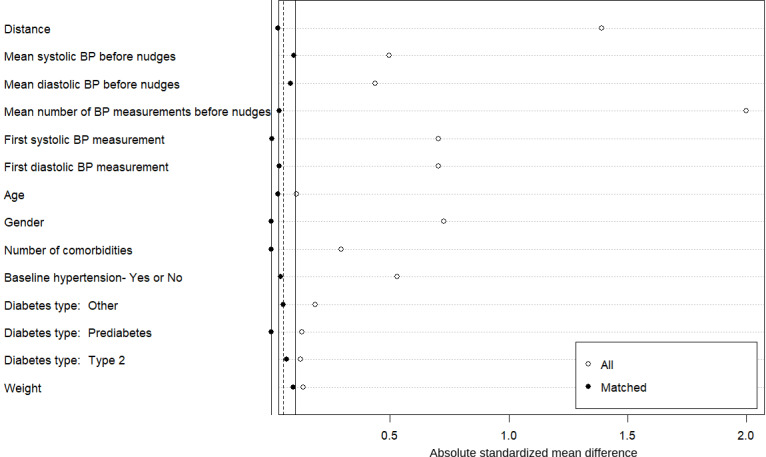
Plot presenting the efficacy of the matching procedure for balancing the normal blood pressure (BP) cohort. Distance refers to the standardized difference in the propensity scores between the treatment and the control after matching. A caliper of 0.1 SD is reached for all parameters.

In this study, the propensity scores were calculated for each participant using the “matchit()” function from the R package *matchit*, and the distance metric used was based on logistic regression using a 1:1 ratio between the 2 study groups: 148 users in each group from the high BP cohort and 56 users in each group from the normal BP cohort. We applied nearest-neighbor matching with a caliper width of 0.1 SDs of the propensity score using logistic regression of the intervention on the covariates. Participants without suitable matches were excluded from the analysis. Figures3  4 present the efficacy of the matching procedures for balancing the groups.

### Analytic Approach

A classical linear longitudinal model assumes a single-slope growth pattern for changes in an outcome variable across time. In contrast, piecewise‐based mixed‐effects models allow flexibility in the modeling of variable change trajectories across time [45]. Here, a piecewise mixed-effects model assessed differences in SBP and DBP in 2 segments: before and after the nudge messages were sent. The piecewise model allowed the data to exhibit different linear trends over their different regions.

In the intervention group, user measurements were centered around receiving the first nudge message in the high BP cohort and the normal BP cohort. In the control group, user measurements were centered around the time of the cluster of events that were the same as in the intervention group. For the high BP cohort, data from 3 months prior to and 3 months following the intervention were included in the analysis. For the normal BP cohort, the analysis included data from 3 months prior to and 6 months following the intervention to assess the sustainability of normal BP events over the longer term.

A piecewise-based mixed-effects model was fitted, modeling temporal changes of the monthly average SBP and DBP for the 2 groups in each cohort (high and normal BP). For the high BP cohort, the monthly average of the high-reading percentage was also modeled. The piecewise cutoff point for the model was set for month 0 (the month that the messages were sent), assuming a change in the time-related monthly average BP between the groups by the included interaction terms between the 2 time trajectories and the groups. Another piecewise mixed-effects model was evaluated only in the high BP cohort to model the effect of the interaction between the 2 time periods and lifestyle activities on the monthly average SBP. We compared three random effect specifications: (1) random intercepts only, (2) random slopes and intercepts, and (3) uncorrelated random effects. Model comparison using the Bayesian information criterion supported the random intercept–only structure that was used in the models. All piecewise mixed-effects models were fitted using restricted maximum likelihood estimation via the lme4 package in R version 4.3.1. Convergence was assessed using gradient tolerance <0.002 and confirmed by positive definite Hessian matrices. Model assumptions were validated through Diagnostics for Hierarchical Regression Models–simulated residual diagnostics and examination of random effect distributions. Next, we used a simple slope analysis to interpret the interaction between the outcomes and the groups. The same statistical framework was applied to test the moderating effect of lifestyle activities on SBP in the high BP cohort. All coefficients from the piecewise mixed-effects models represent the rate of change in the outcome variable per month. For BP outcomes (SBP and DBP), coefficients are expressed in mm Hg per month, indicating the average monthly change during each time period.

## Results

### Users

In total, 408 users were included in the study. The high BP cohort had 296 users, including 186 (62.8%) men and 110 (37.2%) women. In this cohort, the average user age was 63.9 (SD 10.0) years, and the average BMI was 32.2 (SD 6.9). Of the 296 users, 196 (66.2%) reported having one or more comorbidities. The normal BP cohort had 112 users, including 30 (26.8%) men, 49 (43.8%) women, and 33 (29.4%) others. In this cohort, the average user age was 55.1 (SD 11.6) years, and the average BMI was 32.0 (SD 10.1). Of the 112 users, 37 (33.0%) reported having one or more comorbidities.

Sensitivity analysis comparing complete case analysis (n=237) with imputed results demonstrated robust findings. In complete cases, the intervention group showed a decrease in monthly SBP of −1.73 mm Hg (95% CI −2.45 to −1.01; *P*<.001), which closely aligned with the primary imputed analysis results (−1.86 mm Hg; *P*<.001). Similar consistency was observed for DBP and high-reading percentage outcomes. The convergence of estimates across missing data approaches, combined with the separation of imputed covariates from observed outcomes, provides strong evidence for the validity of our findings.

### Study 1: Association of the Digital Intervention With Improvements in High BP Levels

**Figure 5.**
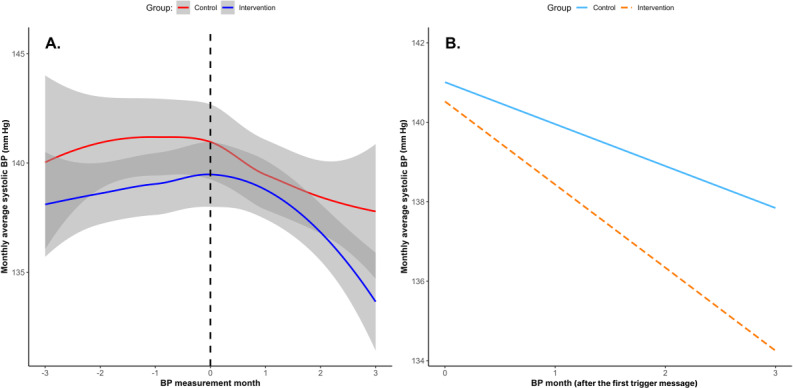
(A) Monthly average systolic blood pressure (BP) change over time. The dotted line represents the month when the first message was sent. (B) Simple slope analysis of the monthly average systolic BP during the period after the first nudge message was sent (moderated by group).

The results from the piecewise mixed-effects model revealed differences in the trajectories of the monthly average SBP between the intervention and control groups, as illustrated in Figure 5A. The intraclass correlation coefficient indicated that 62% of the total variance in SBP was attributable to between-subject differences.

A significant interaction effect was observed following the delivery of the first nudge message (B=−1.04; *P*=.04), while no significant interaction was detected in the preceding period (B=−0.21; *P*=.67). The intervention group (n=148) demonstrated a significant decrease in the monthly average SBP during the 3 months following the nudge message (−2.09 mm Hg/month, 95% CI −2.77 to −1.41; *P*<.001), with a total reduction of 6.27 mm Hg over the 3-month period. The control group (n=148) also exhibited a significant reduction in the monthly average SBP (−1.06 mm Hg/month, 95% CI −1.83 to −0.28; *P*=.007), with a total reduction of 3.18 mm Hg over 3 months. The differential effect between groups (1.04 mm Hg/month) represents the additional benefit attributable to the digital nudges (Figure 5B).

**Figure 6.**
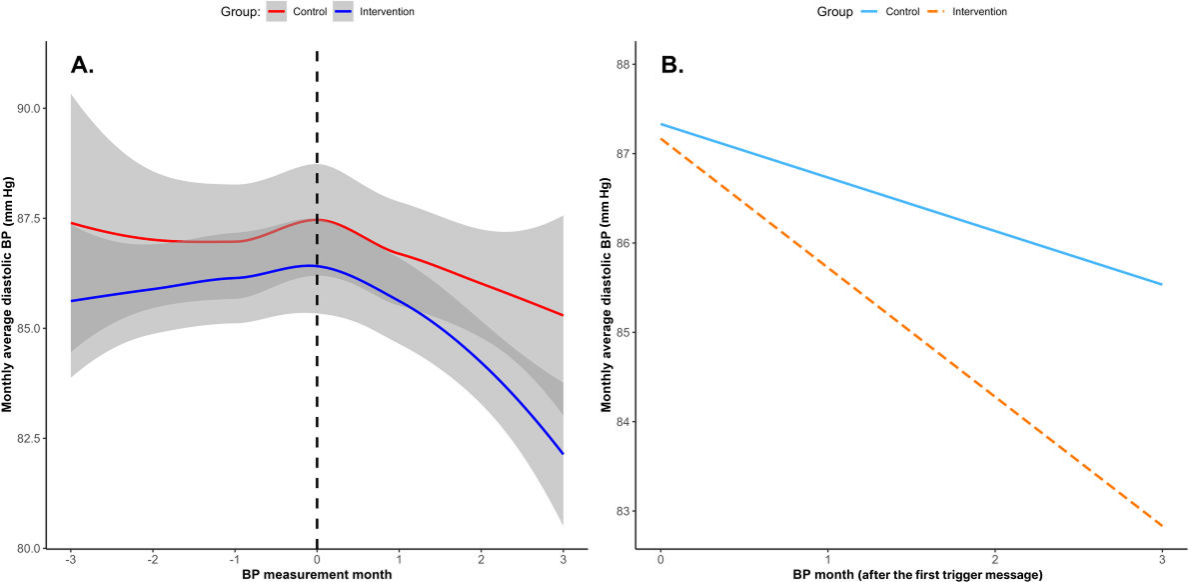
(A) Monthly average diastolic blood pressure (BP) change over time. The dotted line represents the month when the first message was sent. (B) Simple slope analysis of the monthly average diastolic BP during the period after the first nudge message was sent (moderated by group).

The results of the piecewise mixed-effects model highlighted differences in the trajectories of the monthly average DBP between the groups, as shown in Figure 6A. The intraclass correlation coefficient indicated that 66% of the total variance in DBP was attributable to between-subject differences.

A significant interaction effect was observed during the period after the first nudge message was sent (B=−0.85; *P*=.02), while the interaction effect was not significant in the preceding period (B=−0.17; *P*=.61).

The intervention group exhibited a significant decrease in the monthly average DBP within the 3 months following the nudge message (−1.45 mm Hg/month, 95% CI −1.91 to −0.98; *P*<.001), with a total reduction of 4.35 mm Hg over the 3-month period. The control group also showed a significant reduction in the monthly average DBP (−0.60 mm Hg/month, 95% CI −1.13 to −0.07; *P*=.03), with a total reduction of 1.80 mm Hg over 3 months. The differential effect between groups (0.85 mm Hg/month) indicated that the intervention group experienced an additional 2.55 mm Hg reduction in DBP attributable to the digital nudges over the 3-month period (Figure 6B).

**Figure 7.**
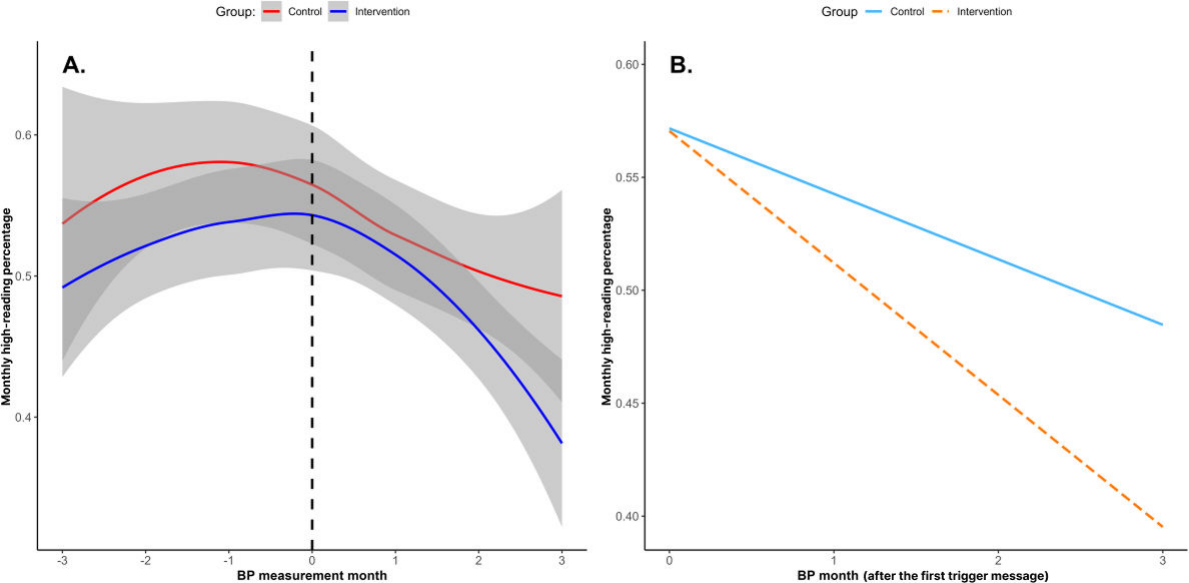
(A) Monthly average high-reading percentage change over time. The dotted line represents the month when the first message was sent. (B) Simple slope analysis of the monthly average high-reading percentage during the period after the first nudge message was sent (moderated by group).

Another piecewise mixed-effects model was used to assess differences in the trajectories of the monthly average high-reading percentage between the groups, as illustrated in Figure 7A. The intraclass correlation coefficient indicated that 54% of the total variance in the monthly average high-reading percentage was attributable to between-subject differences.

A significant interaction effect was observed during the period after the first nudge message was sent (B=−0.029; *P*=.04), while no significant interaction was found in the prior period (B=0.0005; *P*=.97). The intervention group exhibited a significant decrease in the monthly high-reading percentage in the 3 months following the nudge message (−5.8 percentage points/month, 95% CI −7.7 to −4.0; *P*<.001), with a total reduction of 17.4 percentage points over the 3-month period. The control group also showed a significant reduction in the monthly high-reading percentage (−2.9 percentage points/month, 95% CI −5.0 to −0.8; *P*=.008), with a total reduction of 8.7 percentage points over 3 months. The differential effect between groups (2.9 percentage points/month) indicated that the intervention group experienced an additional 8.7 percentage point reduction in high readings attributable to the digital nudges over the 3-month period (Figure 7B).

Finally, a piecewise mixed-effects model was applied exclusively to the intervention group to evaluate the interaction between the 2 time periods and lifestyle activities in terms of the monthly average SBP. The results revealed a significant interaction effect between the number of lifestyle activities (operationalized as the sum of meal logs, carbohydrate intake, calories burned, and recipe finder activities in each month) and the monthly average SBP during the postmessage period (B=−0.12; *P*=.004), whereas no significant effect was observed in the premessage period (B=0.13; *P*=.33).

**Figure 8.**
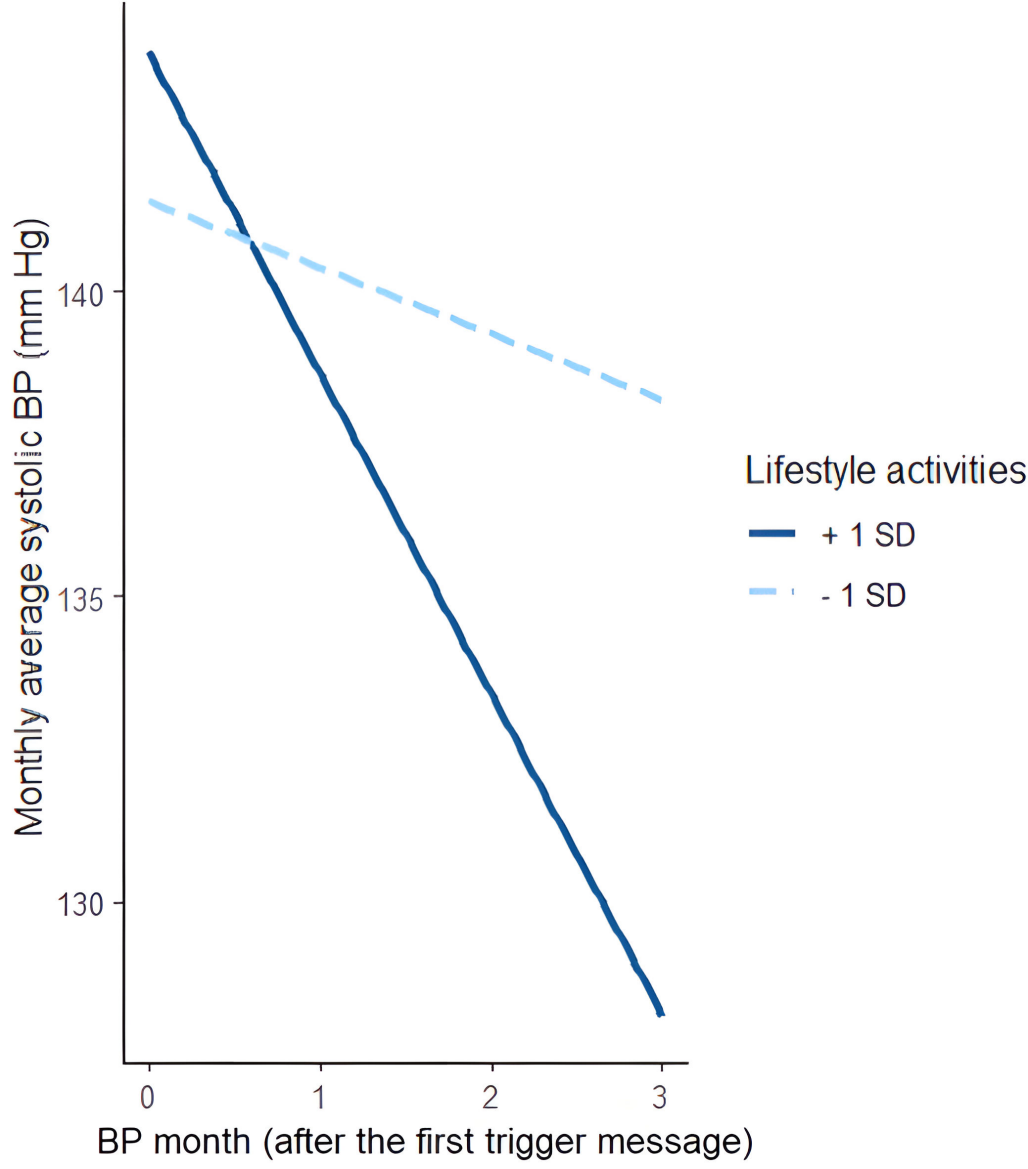
Simple slope analysis of the monthly average systolic blood pressure (BP) during the period after the first nudge message was sent (moderated by the number of lifestyle activities).

A simple slope analysis indicated that users with a higher monthly number of lifestyle activities experienced significant reductions in the monthly average SBP (B=−5.27; *P*<.001), while users with a below-average monthly number of lifestyle activities showed no significant changes (B=−1.08; *P*=.23) (Figure 8).

### Study 2: Association of the Digital Intervention With the Sustainability of Normal BP Levels

**Figure 9.**
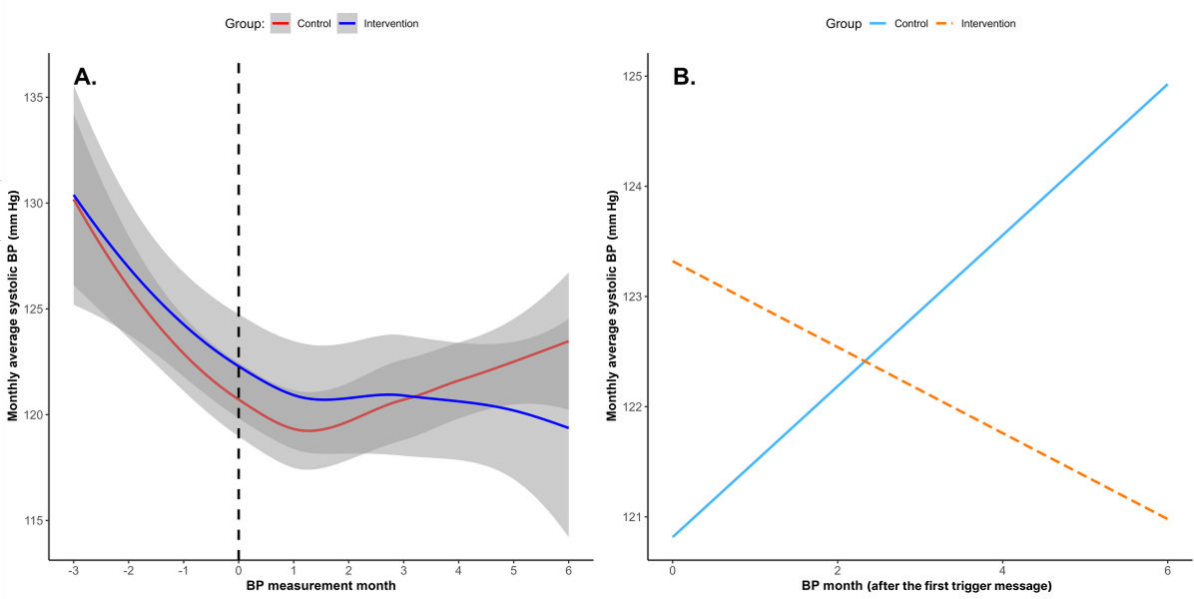
(A) Monthly average systolic blood pressure (BP) change over time. The dotted line represents the month when the first message was sent. (B) Simple slope analysis of the monthly average systolic BP during the period after the first nudge message was sent (moderated by group).

The results of the piecewise mixed-effects model highlighted differences in the trajectories of the monthly average SBP between the groups, as shown in Figure 9A. The intraclass correlation coefficient indicated that 36% of the total variance in SBP was attributable to between-subject differences.

A significant interaction effect was observed in the 6-month period following the first nudge message (B=−1.08; *P*=.02), whereas no significant interaction effect was found in the period prior to the message (B=1.10; *P*=.32).

A simple slope analysis revealed that in the months following the messages, the intervention group (n=56) sustained stable BP levels with no significant change in the monthly average SBP (−0.39 mm Hg/month, 95% CI −1.08 to 0.30; *P*=.27). In contrast, the control group (n=56) exhibited a significant increase in the monthly average SBP (0.69 mm Hg/month, 95% CI 0.08 to 1.29; *P*=.03), with a total increase of 4.14 mm Hg over the 6-month period. The differential effect between groups (1.08 mm Hg/month) indicated that the digital nudges prevented an increase of approximately 6.48 mm Hg in SBP over the 6-month follow-up period compared to the control group (Figure 9B).

**Figure 10.**
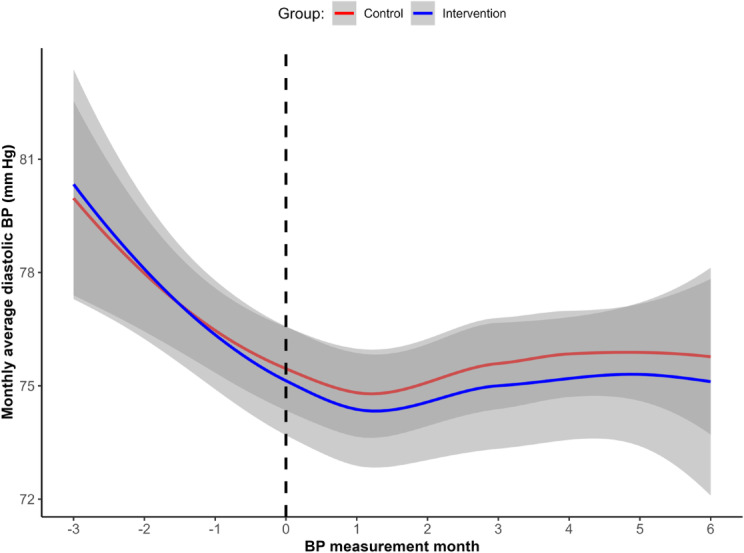
Monthly average diastolic blood pressure (BP) change over time. The dotted line represents the month when the first message was sent.

Another piecewise linear mixed-effects model was used to examine the differences in the trajectories of the monthly average DBP between the groups over the 3 months preceding and the 6 months following the first nudge message (Figure 10). The intraclass correlation coefficient indicated that 38% of the total variance in DBP was attributable to between-subject differences. The results revealed that neither group showed significant changes in DBP during the 6-month period following the intervention. The interaction effects between groups were not significant either before or after the messages (before the messages: B=−0.31; *P*=.64; after the messages: B=−0.13; *P*=.63), indicating no differential effect of the digital nudges on BP in the normal BP cohort. Both the intervention and control groups maintained stable DBP levels throughout the study period (Figure 10).

## Discussion

### Principal Findings

This retrospective study evaluated the effectiveness of a digital intervention aimed at helping individuals with high BP to regulate their levels by providing tailored digital support at the right time. This support was customized to individual needs and was compared to a matched control group that did not receive it. The findings revealed that users with higher engagement in lifestyle activities experienced significant reductions in their monthly average SBP. Additionally, the study suggested the critical role of positive support through messaging for maintaining normal BP levels. This support may enhance an individual’s overall well-being and encourage positive behaviors that help sustain healthy BP levels over time and thus may support long-term hypertension management.

Our study used propensity score matching for the control group and used a piecewise mixed model as a statistical framework to describe the nonlinear behavior in BP levels, comparing 2 groups over time.

In the high BP cohort, our analysis indicated that before the intervention phase, the high readings in both groups demonstrated similar trajectories in terms of BP levels. However, after the start of the nudges, the BP levels in the intervention group significantly reduced, while the BP levels in the control group experienced a less pronounced effect and showed only a slight or less significant change. In the normal BP cohort, no significant interaction effect was found in the period prior to the positive feedback messages, while after the positive feedback messages were delivered, the intervention group sustained the reduction in their monthly average SBP, while the control group exhibited a significant increase in their monthly average SBP in the following 6-month period.

Robust statistically significant effects were found for improved diet, aerobic exercise, alcohol and sodium restriction, and fish oil supplements (mean reductions in SBP of 5.0, 4.6, 3.8, 3.6, and 2.3 mm Hg, respectively), with corresponding reductions in DBP [46].

Hypertension is a major cause of cardiovascular disease and kidney disease, and BP control reduces the risk of these complications [47,48]. Despite the availability of effective hypertensive medications, BP treatment and control rates remain low [48]. Self-management education, including user education, self-monitoring of clinical measurements, lifestyle modifications (such as a healthy diet, physical activity, weight management, smoking cessation, and alcohol reduction), and support for medication adherence, has been widely used for BP management [49,50]. Providing information about hypertension and its treatment, along with home BP monitoring and feedback, has been incorporated into effective interventions, demonstrating that supported self-management can improve BP control [50].

Healthy lifestyle and diet are associated with significant reductions in the risks of obesity, type 2 diabetes, high BP, and cardiovascular diseases [51]. Diet plays a crucial role in BP regulation through multiple mechanisms such as improved endothelial function, enhanced pressure natriuresis, and reduced oxidative stress [52]. Dietary management is considered to be an effective treatment method for hypertensive users as diet has a direct link with BP regulation [53,54]. Previous studies have suggested that lowering sodium intake mitigates hypertension symptoms significantly by lowering BP [55]. Conversely, increased sodium intake activates the hormonal mechanisms implicated in the pathogenesis of hypertension [55].

Recent studies have identified potential mechanisms by which physical activity enhances vascular function [56]. Regular exercise enhances vascular health through improvements in endothelial function. Specifically, exercise training in individuals with stable coronary artery disease has been shown to improve agonist-mediated, endothelium-dependent vasodilatory capacity [56]. Aerobic and resistance exercises have physiologically meaningful effects on endothelial function [57], increasing both the gene and protein expressions of endothelial nitric oxide synthase and boosting nitric oxide production in patients with coronary artery disease [51,58], which can result in arterial vasodilation and reduced peripheral resistance. This mechanism directly contributes to lower BP [59].

This study demonstrated that the use of automated data-driven nudges for interventions in hypertension can significantly influence BP levels over time. Digital messages may be effective for BP control by promoting lifestyle changes and improving medication adherence. Such interventions can encourage users to not only adopt healthier habits but also stay adherent to prescribed treatments or seek medical consultation for therapy adjustments in different populations, including low- and middle-income populations [60].

One of the broad mechanisms underlying the benefits of digital health tools is biofeedback to improve monitoring and management [24]. Digital health nudges impact the implementation and adherence to guideline-driven, evidence-based nonpharmacological strategies for reducing BP. This includes raising the awareness of how to properly measure BP at home, understanding BP categories and risk levels, promoting a healthy diet, reducing dietary salt intake, encouraging physical activity, managing stress, improving sleep hygiene, and addressing smoking and alcohol consumption [24,29,33,46,61]. The different components may also interact. For example, both stress reduction and decreased salt intake could contribute to better sleep quality [62].

The DASH diet has been reported to significantly lower SBP at every sodium level and significantly lower DBP at the high and intermediate sodium levels [33]. Through feedback messages, users receive guidance on proper medication use, including tips to improve adherence. Adherence is important, and high adherence to antihypertensive medication is associated with higher odds of BP control. Nonadherence to cardioprotective medication increases a user’s risk of death from 50% to 80% [63]. Multiple factors contribute to adherence levels, stemming from individual, provider, and health care system elements, which often interact with each other [64]. Developments in the field of digital adherence monitoring and management are rapidly evolving to different cutting-edge solutions ranging from smartphone apps to smart devices. Technical and social innovations could lead to improved medication adherence and better user outcomes [34,65]. Self-monitoring of BP using a digital home BP monitoring device is another essential component of digital health in users with hypertension [23,29]. Use of out-of-office BP monitoring is preferred over office BP monitoring for the diagnosis and management of hypertension in major hypertension guidelines [29].

In the intervention presented in this manuscript, decisions concerning when and how to provide support are intervention-determined rather than user-determined. Tailoring variables were determined through active assessment of BP levels, which required user engagement. Users included in the study received prompts about their BP status when high readings (elevated and stage 1 or stage 2) were detected on 3 separate days within a 7-day period.

Real-time behavioral support that directly addresses user needs, where the content or timing of support is adapted and based on system-collected input and is triggered by the system, has been shown to be effective in digital health interventions [25]. The use of mHealth technology has been recommended as portable devices make intervention delivery more interactive and responsive by sending feedback messages in real time based on the user’s state (eg, to promote physical activity and reduce sedentary behavior) [66,67]. Progress in digital health technology has enabled the design of interventions that aim to deliver behavior change support in real time, and this is matched to when users most want or need an intervention or when they are at risk. One of the common behavior change techniques is prompts/cues or feedback on behavior and action planning [25,68]. Digital health interventions, such as remote BP monitoring and tailored feedback, have been shown to be associated with significant reductions in BP levels among diverse populations [69]. Importantly, the findings of this study align with the results of previous meta-analyses, showing that individuals who received digital nudges experienced a greater reduction in SBP compared to those in the control group [22,69-71]. One of the most successful mHealth interventions combined the features of tailored messages, interactive communication, and multifaceted functions [71]. The findings of our study, which compared an intervention group to a control group using the same platform with or without the intervention, highlight the benefits of data-driven nudges that deliver real-time messages. The results suggest that adaptive, system-triggered support can effectively assist individuals in managing their BP. These outcomes could inform the development of an artificial intelligence–driven platform, which could act as a precise lifestyle guide for individuals with hypertension. Such a system could integrate BP monitoring with lifestyle data and leverage personalized machine learning models to assess the individual impact of various factors on BP. Previous programs typically provided users with remote monitoring devices and paired them with health coaches, but did not account for the individual impact of lifestyle factors on BP, which can vary due to physiological differences [72]. In addition, physicians are often unable to optimally counsel patients on lifestyle modifications or personalize their guidance due to time constraints related to workload [73,74]. BP and lifestyle data collected from digital health platforms may allow us to view trends and make personalized recommendations to users. Personalized recommendations were found to be more useful compared to generic recommendations [72]. We believe that the combination of personalized guidance, ease of adherence, and motivational reinforcement contributed to high engagement and improved BP outcomes.

Our study also sheds light on the effect of positive feedback on normal BP levels in the long term, indicating that integrating positive behavioral principles can help users build resilience, improve motivation, and enhance user engagement. Positive feedback in digital health nudges has been shown to enhance user engagement and lead to better clinical outcomes in BP management through BP monitoring with supportive digital applications, which can increase user awareness and facilitate behavioral change, resulting in improved BP control [23].

Previous studies highlighted the importance of user empowerment tools, such as user-reported outcome measures and shared decision-making processes, in managing chronic diseases [75]. The integration of positive feedback within digital health interventions plays a crucial role in motivating users to adhere to their management plans, as demonstrated in previous studies [76]. We believe that incorporating positive feedback on normal BP levels enhances user motivation, fosters a hopeful mindset, and reinforces user confidence in making improvements. Additionally, recognizing and leveraging users’ strengths can boost self-efficacy and encourage active engagement in their treatment plans [77]. In contrast, users who do not receive positive reinforcement may be less aware, potentially leading to increased BP levels over time.

By incorporating these positive psychology messages into clinical care, health care providers can support users in a holistic way, improving not only clinical outcomes but also users’ quality of life and overall health management.

Additional research is needed to explore the best way to integrate user-determined features into a system-driven intervention, balancing structured, externally initiated support with individual autonomy in managing hypertension and other metabolic chronic conditions [78].

### Limitations

This study had several limitations. As with all retrospective real-world data analyses, the groups were not randomly assigned and treatment protocols were not prescribed, creating challenges in drawing causal inferences. Statistical modeling can address some difficulties in comparing groups and allow quasi-causal inferences; however, unmeasured variables may impact group balance.

While we carefully selected covariates for propensity score matching based on clinical relevance and prior literature, unmeasured confounding remains possible. Factors, including socioeconomic status, health literacy, dietary habits, stress levels, and intrinsic motivation for health behavior change, were not directly measured and could influence outcomes. Critically, this study lacked information on concurrent antihypertensive medication use and changes, which could significantly impact BP trajectories. Several aspects of our design helped mitigate these concerns. Matching on baseline BP measurement frequency partially captured health engagement, and the balance achieved on measured covariates (Figures3  4) reduced the likelihood of substantial unmeasured confounding. Moreover, as the intervention and control groups experienced identical BP event patterns, unmeasured factors likely affected both groups similarly. Nevertheless, unmeasured characteristics that were differentially distributed between the groups could have influenced our effect estimates, an inherent limitation of a retrospective observational design. The COVID-19 pandemic was a major uncontrolled event separating the control and intervention periods, introducing a potential secular trend bias. Broader societal changes, such as increased health awareness and health care access, may have influenced user behavior and engagement patterns independent of the digital nudges.

The study population was limited to individuals who self-selected the use of the Dario system, indicating pre-existing health care engagement. Users who opened and read intervention messages may have been particularly motivated to change. However, our inclusion criteria ensured that both groups showed evidence of engagement with hypertension management, with no significant differences in BP measurement frequency between the groups, suggesting that motivation may not be the primary differentiating factor.

We acknowledge that external validity may be limited to health-engaged, technologically enabled populations, and the results may not be generalizable to those with limited access or comfort with digital tools. However, the growing use of smartphones and digital health across diverse groups suggests cautious optimism for broader applicability. Future research should focus on strategies to engage lower-motivation users and assess effectiveness in more diverse, underserved populations.

Additionally, Bluetooth-connected devices operating outside electronic medical records face adoption challenges, particularly for users with limited health technology literacy. The successful device adoption in our study population may limit generalizability. Nevertheless, the clinical benefits observed align with growing evidence that digital health interventions can achieve meaningful outcomes even without full electronic medical record integration, particularly when combined with behavioral support features like our automated nudge system.

Our temporal analysis design focused on monthly intervals over a 3-month (high BP cohort) or 6-month (normal BP cohort) period before and after the intervention. While relationships could potentially be investigated at daily or weekly scales, tracking such granular changes is difficult in real-world studies. Monthly average BP provides robust estimates of sustained intervention effects and aligns with clinical practice guidelines while providing sufficient statistical power to detect clinically meaningful effects. However, this approach cannot capture short-term variability patterns, including intraday fluctuations, morning surges, or circadian patterns that have independent prognostic values for cardiovascular outcomes. Future studies with higher-frequency measurements could investigate whether digital interventions affect these BP variability parameters.

Critically, we cannot adjust for time-varying confounders over the 6-month study period. Our piecewise mixed-effects model accounts for individual trajectories, but it cannot control postbaseline medication changes, clinical visits, or seasonal BP variations. The use of historical controls versus intervention recipients can introduce a potential secular trend bias. An additional limitation of this study is the lack of stratified analysis on the intensity or frequency of digital nudges and their specific effects on BP outcomes. While the intervention demonstrated overall effectiveness, we did not assess whether higher or lower exposure to messaging would yield differential impacts on SBP or DBP outcomes. Future research should investigate the differential effectiveness of nudge intensity and timing, using experimental or adaptive designs, which will lead to more precise and effective hypertension management at scale.

Finally, available demographic and medical data were limited. Although no differences existed between the groups in age, gender, median household income, diabetes type, weight, or comorbidities, uncontrolled bias from other demographic or medical factors remains a possibility. Future studies should incorporate comprehensive medication data to better isolate digital intervention effects and explore strategies to reduce adoption barriers while maintaining demonstrated clinical effectiveness.

### Conclusions

This study provides evidence suggesting that digital health data–driven nudges may help support BP management by delivering real-time, adaptive behavioral support. Users who engaged more actively with lifestyle interventions experienced greater reductions in SBP, indicating a potential association between engagement and improved outcomes. Our findings highlight the significance of integrating positive messaging into digital health solutions. Positive feedback on normal BP levels may have encouraged users to maintain healthy behaviors, reinforcing motivation and self-efficacy. In contrast, those who did not receive positive feedback exhibited an upward trend in BP levels over time. This underscores the value of continuous engagement and reinforcement strategies in sustaining long-term hypertension management.

The use of a digital health platform enabled real-time monitoring, while data-driven nudges further enhanced user engagement by prompting timely self-monitoring, reinforcing healthy behaviors, and supporting adherence to lifestyle modifications and antihypertensive therapy. The application of machine learning models and artificial intelligence–driven platforms could further enhance the personalization of digital health interventions, tailoring recommendations based on individual responses to behavioral and physiological data.

### Future Research Directions

Despite the promising results, additional research is required to refine digital health interventions, ensuring they are both system-driven and adaptable to user preferences. Future studies should explore the optimal integration of user-determined features within structured, externally initiated interventions. Additionally, expanding these approaches to other cardiometabolic conditions could further validate the effectiveness of digital health tools in managing chronic diseases. By leveraging personalized recommendations, behavioral reinforcement, and positive feedback, digital health platforms have the potential to transform hypertension care, empowering individuals to take an active role in their health and achieve sustainable improvements in BP control.

## Abbreviations

AHA: American Heart Association
BP: blood pressure
DASH: Dietary Approaches to Stop Hypertension
DBP: diastolic blood pressure
JITAI: just-in-time adaptive intervention
SBP: systolic blood pressure
SMD: standardized mean difference
